# Overexpression of PIN1 Enhances Cancer Growth and Aggressiveness with Cyclin D1 Induction in EBV-Associated Nasopharyngeal Carcinoma

**DOI:** 10.1371/journal.pone.0156833

**Published:** 2016-06-03

**Authors:** Meng Xu, Chartia Ching-Mei Cheung, Chit Chow, Samantha Wei-Man Lun, Siu-Tim Cheung, Kwok-Wai Lo

**Affiliations:** 1 Department of Oral Pathology, Guangdong Provincial Key Laboratory of Stomatology, Hospital of Stomatology, Sun Yat-sen University, Guangzhou, People’s Republic of China; 2 Department of Anatomical and Cellular Pathology, State Key Laboratory in Oncology in South China, Prince of Wales Hospital, The Chinese University of Hong Kong, Shatin, N.T., Hong Kong; 3 Department of Surgery, The Chinese University of Hong Kong, Shatin, N.T., Hong Kong; 4 Li Ka Shing Institute of Health Science, The Chinese University of Hong Kong, Shatin, N.T., Hong Kong; Gustave Roussy, FRANCE

## Abstract

**Background:**

Nasopharyngeal carcinoma (NPC) is a peculiar Epstein Barr virus (EBV)-associated malignancy that is prevalent in South-East Asia. Peptidyl-prolyl *cis-trans* isomerase NIMA-interacting 1 (PIN1) isomerizes specific phosphorylated amino acid residues, which makes it an important regulator in cell survival and apoptosis. In this study, we investigated the contribution made by PIN1 in NPC tumorigenesis and PIN1’s potential role as a therapeutic target.

**Methods:**

The expression of PIN1 was examined in a panel of NPC cell lines, xenografts and primary tumors. The functional roles of PIN1 in NPC cells were elucidated by the knockdown and overexpression of PIN1 in *in vitro* and *in vivo* nude mice models by siRNA and lenti-viral transfection, respectively. The antitumor effects of the PIN1 inhibitor Juglone in NPC cells were also evaluated.

**Results:**

We revealed the consistent overexpression of PIN1 in almost all EBV-associated NPC cell lines, xenografts and primary tumors. PIN1 suppression was capable of inhibiting cyclin D1 expression and activating caspase-3 in NPC cells. It positively regulated NPC cell proliferation, colony formation and anchorage-independent growth. The inhibition of PIN1 suppressed tumor growth *in vitro* and *in vivo*.

**Conclusions:**

This study demonstrates the oncogenic role of PIN1 in NPC tumorigenesis, and shows that its overexpression can enhance tumor cell growth via the upregulation of cyclinD1. Our findings inform the development of novel treatments targeting PIN1 for NPC patients.

## Introduction

Nasopharyngeal carcinoma (NPC) is a distinctive type of head and neck carcinoma that arises from the epithelial cells covering the surface and lining of the nasopharynx. This malignancy exhibits a distinct ethnic and geographical distribution, and is particularly prevalent in Southern China, where almost all cases are nonkeratinizing carcinomas associated with EBV infection [1, 2]. This association with EBV, in addition to the existence of multiple genetic aberrations [3], increases the complexity of NPC research. The poor results achieved by conventional chemo-radiotherapy for patients with advanced loco-regional diseases and distant metastases represent a therapeutic challenge [4]. Given the high relapse and metastasis rates, it is of utmost importance that alternative therapeutic approaches be sought out and developed.

Cancer development involves a complex array of aberrant signaling pathways that fundamentally results in uncontrolled cell proliferation controlled by proline-directed phosphorylation [5]. PIN1 is a highly conserved enzyme that binds to and isomerizes specific phosphorylated serine or threonine residues preceding proline (Ser/Thr-Pro) bonds. It induces conformational changes in certain proteins that prompt changes to their properties, including catalytic activities, subcellular localization, protein–protein interactions and turnover rate [5, 6]. Thus, PIN1 is considered an important regulator in cellular processes, such as cell cycle regulation, cell signaling, transcription and splicing, DNA-damage responses, cell survival and drug resistance [5, 7, 8].

Apart from the importance of PIN1 in modulating the activation of various signaling molecules, it has also been shown to stabilize viral oncoproteins such as the human T-cell leukemia virus type I Tax protein [9]. Moreover, PIN1 has been reported to be involved in the functioning of several viruses, including Kaposi’s sarcoma-associated herpes virus [10], human immunodeficiency virus type I [11, 12] and the hepatitis C virus [13]. Interestingly, it has also been demonstrated to interact with EBV-encode protein [14], although information on its role in EBV-associated malignancy has proven scarce.

This study aimed to elucidate the role of PIN1 in the development of NPC which is consistently associated with EBV infection. By investigating the effects of PIN1 expression on EBV-associated NPC, we revealed its contribution to tumor cell growth and tumorigenesis.

## Materials and Methods

### Cell lines, xenografts and primary tumors

Three NPC cell lines C666-1 (a naturally EBV-associated, undifferentiated type of NPC) [15], HK1 (an EBV-negative, well-differentiated type of NPC) [16], HK1-EBV (HK1 infected with EBV) [17], immortalized normal nasopharynx epithelial cell lines (NP69 and NP460) [18] established in our laboratories were used in this study. The cervical cancer cell line HeLa was obtained from American Type Culture Collection (ATCC). Two EBV-positive NPC xenografts—xeno-666 [15], xeno-2117 [19], C15 and C17 [20] were also included. Xen-666 and xeno-2117 were established by our NPC group as described previously [15, 19]. C15 and C17 were obtained from Prof. Pierre Busson who established these xenografts [20]. For the immunohistochemical (IHC) staining study, 70 archival formalin-fixed paraffin embedded (FFPE) primary NPC biopsies were recruited from the tissue bank of Department of Anatomical and Cellular Pathology at the Prince of Wales Hospital, The Chinese University of Hong Kong (CUHK). The study protocol was approved by the Clinical Research Ethics Committee of the CUHK. All of the specimens were histologically evaluated as undifferentiated or poorly differentiated carcinomas and confirmed to be EBV-positive, as determined by EBER *in situ* hybridization [21]. The patients’ characteristics are presented in Table 1.

**Table 1:** Characteristics of NPC patient specimens recruited for IHC study.

*Variables*	*No*. *of patients*
**Age (years)**	
≤ 50	41
> 50	29
**Gender**	
Male	57
Female	13
**Clinical stage**	
Early (Stages 1 and 2)	21
Late (Stages 3 and 4)	49
**RecurrenceRecurrence/distant metastasis**	
Absent	53
Present	17

### Transfection and drug treatments

HK1, HK1-EBV, C666-1 and HeLa cells were cultured in RPMI1640 medium supplemented with 10% fetal bovine serum and 1% L-glutamine (Life Technologies). The NP69 cells were cultured in keratinocyte serum-free medium (KSFM) with bovine pituitary extracts and recombinant epidermal growth factor (Invitrogen). All of the cells were incubated in a humidified atmosphere with 5% CO_2_ at 37°C.

Two Silencer Select validated siRNAs targeting PIN1 (siPin1 544 and siPin1 545) and universal negative control siRNA (Life Technologies) were transiently transfected into C666-1 using Lipofectamine^TM^ 2000 Transfection Reagent (Invitrogen) according to the manufacturer’s protocols. Cells were collected at indicated time points for further analysis. MG132 (Calbiochem) was applied to HK1 and NP69 cells (at 10 and 15 μM and 5 and 10 μM, respectively) to study the proteasome degradation of PIN1. The PIN1 inhibitor Juglone (Calbiochem) was used to study the effect of PIN1 inhibition on C666-1 cells (at 2, 4, 6 and 8 μM). Treated cells were collected and stored at -80°C until use. To determine the IC50 of Juglone, the cells were seeded into 96-well plates and treated with different Juglone dosages (0.03–20 μM) for 24 hours. Cell viability was then determined by a WST-1 assay.

### Establishing stable PIN1-overexpressing cell lines

The pCDH and pCDH-PIN1 plasmids were a gift, generously provided by Dr. Roberta Pang, Department of Surgery, The University of Hong Kong. A lentiviral system was used to establish the stable PIN1-overexpressed NP69 cell line. The pCDH lentiviral vector was packed using the third-generation lentivirus system, according to the Lenti Starter kit manufacturer’s protocols (System Biosciences). Viral supernatant (culture media) was harvested from 293TN producer cells 48 hours after transfection. The virus (with a pCDH or pCDH-PIN1 vector and 10 μg pPACKH1-plasmid mix) was collected and transduced with TransDuxand into NP69 cells. PIN1 expression was confirmed by quantitative polymerase chain reaction (qPCR) and Western blotting. Green fluorescence protein (GFP) reporter expression was visualized under fluorescence microscope to ensure the presence of the transduced DNA.

### Reverse transcription (RT) and quantitative polymerase chain reaction (qPCR)

RNA samples prepared by TRIZOL and phenol-chloroform extraction were subjected to reverse-transcription using MultiScribe Reverse Transcriptase (Invitrogen). The cDNA obtained was used for real-time qPCR via SYBR Green Master Mix (Applied Biosystems), according to the manufacturer’s protocols. The primers used in this study are listed in Table 2. The PCR reactions were performed using the 7500 Fast Real-Time PCR system (Applied Biosystems). Each sample was analyzed in triplicate. The expression of target genes was normalized against the housekeeping gene β-actin using the 2^[-ΔΔCT]^ method.

**Table 2:** Quantitative PCR primer sequences.

*Gene*	*Forward primer (5’→ 3’)*	*Reverse primer (5’→ 3’)*
PIN1	GGAGGCCCTGGAGCTGAT	AACTGTGAGGCCAGAGACTCAAA
Actin	GTCTTCCCCTCCATCGTG	AGGGGTGAGGATGCCTCTCTT

### Western blotting

The protein samples were separated according to their sizes by electrophoresis, and then electro-transferred to a nitrocellulose membrane using a Bio-Rad Trans-Blot cell. The nitrocellulose membrane was incubated with primary antibodies overnight in 5% non-fat milk or 5% bovine serum albumin. The membrane was then incubated with horseradish peroxidase (HRP)-conjugated secondary antibodies. The target proteins were detected by chemiluminescent substrates (GE Life Science) and the emitted signal was detected on X-ray films (Kodak). The membranes were probed with antibodies against human PIN1 (Calbiochem), ACTIN (Santa Cruz), CYCLIN D1 (Lab Vision), β-catenin, c-JUN, c-JUN (Ser73) and c-JUN (Ser63) (Cell Signaling).

### Immunohistochemical staining

The FFPE specimens were sectioned (4 μm), de-paraffinized and rehydrated for subsequent immunostaining. Following antigen retrieval, endogenous biotin activity was blocked by normal bovine serum and the sections were incubated in primary antibodies (anti-PIN1, Calbiochem) in a moist chamber. The HRP-labeled secondary antibody was applied to the sections and, finally, the 3,3’-diamino-benzidine (DAB) substrate was applied for color development. PIN1 expression and localization was visualized in red and nucleuses were counterstained with hematoxylin, which appeared blue. The slides were then dehydrated and mounted with Permount mounting medium (Fisher Scientific).

### Caspase-3 activity assay

The cell apoptosis of treated and non-treated cells was detected using the CaspACEAssay^TM^ System according to the manufacturer’s protocols (Promega). Wells in duplicates containing blank (no cell extract), negative control (extract from untreated cells), induced apoptosis (extracts from PIN1 inhibitor-treated cells) and cells treated with caspase inhibitor (extracts from PIN1 inhibitor- and Z-VAD-FMK the caspase inhibitor-treated cells) were included in the experiments. The experiments were performed in triplicate and the absorbance of the colors developed was measured at a wavelength of 405 nm by a Perkin Elmer 1420 Multilabel Counter Victor 3 (Perkin Elmer). The caspase-specific activity was calculated according to the manufacturer’s guidelines.

### WST-1 and BrdU assays

The cell proliferation reagent WST-1 (Roche) was used to determine the rate of cell proliferation in treated- and non-treated cells. The treated cells were seeded into a 96-well plate at a density of 3000–8000 cells per well. Cell viability was estimated every 24 hours by WST-1, continuously, for 5 days. The absorbance in each well was measured at a wavelength of 450 nm and normalized using a wavelength of 690 nm, detected by the Victor 3 (Perkin Elmer). Cell proliferation was also measured in terms of DNA incorporation within proliferating cells using the BrdU assay (Cell Proliferation ELISA, BrdU colorimetric Kit, Roche).

### Anchorage-independent growth assay

The treated and non-treated cells were plated in soft agar (base agarose: 1.8% agarose in KSFM; top agarose: 0.9% agarose pre-mixed with cells; 2 mL overlaying KSFM medium) to evaluate their growth in an anchorage-independent manner. The plates were incubated at 5% CO_2_ at 37°C for a month. The colonies were visualized using 0.1% p-iodonitro tetrazolium violet (INT) stain (Sigma-Aldrich), and then counted.

### Colony formation assay

The treated and non-treated cells were seeded at 8X10^3^ cells per well (C666-1) or 1X10^3^ cells per well (NP69) into 6-well plates. The cells were kept at 37°C in an incubator with 5% CO_2_ for 21–28 days. The colonies formed were fixed with methanol and visualized in blue using Giemsa (Sigma-Aldrich) staining. The colonies containing over 50 cells were counted.

### *In vivo* tumorigenicity

To elucidate the *in vivo* tumorigenic effect of PIN1, NP69 cells (treated with siRNAs of PIN1 or with vector control) were subcutaneously inoculated into the flanks of female BALB/c nude mice (nu/nu) (4 mice per group). All mice were anesthesized by 2,2,2-tribromoethanol (Avertin) prior to inoculation. Matrigel (BD Bioscience) was used in the inoculation process for NP69. The mice were inspected daily for tumor formation and the sizes of the tumors formed were recorded. To determine the anti-tumor effect of PIN1 inhibitor *in vivo*, Juglone (0 mg/kg, 0.5 mg/kg, 1.5 mg/kg) was injected intra-peritoneally into nude mice implanted with C666-1 luciferase cells when the tumors had reached their appropriate sizes. Tumor size was recorded daily and the luciferase salt was injected into the nude mice on days 0, 6 and 8 to monitor the changes in tumor size via an IVIS^TM^ 100 Imaging system. All of the mice were sacrificed at the end of the experiments by cervical dislocation and the tumors were preserved for further analysis. No mice were ill, died or required early termination during this study. Ethical approval was obtained from the University Animal Experimentation Ethics Committee (AEEC), CUHK, and the animal license was approved by the Hong Kong Government, Department of Health.

### Luciferase reporter assay

The C666-1 cells at 60% confluence in the 96-well plate were co-transfected with FR-TK-Luc plasmid, PIN1 siRNA and reporter plasmid. After 48 hours of transfection, the cells were lysed with a 1X passive lysis buffer (Promega). The lysates were then transferred to a 96-well plate and luciferase activities were assayed using the Dual Luciferase Reporter Kit (Promega) according to the manufacturer’s protocols. The luciferase signal was measured by Victor 3 (Perkin Elmer) with or without automatic injections. The results were expressed as the ratio of Firefly luciferase activity to Renilla luciferase activity.

### Signaling pathway array

A Cancer 10-Pathway Reporter Luciferase Kit (SA Biosciences) was used to determine signaling pathway aberrations in the treated cells. In brief, reporter plasmids on the Cignal Finder Array Plate were resuspended and mixed with 0.8 μL Fugene HD reagent (Roche) in 100 μL OPTI-MEM medium (Invitrogen). The transfection complex was allowed to form at room temperature for 20 minutes. The treated cells were then collected and reverse-transfected with reporter plasmid at a density of 1.5X10^4^ cells per well. The dual-luciferase reporter assay was conducted after 24 hours of incubation.

### Statistical analysis

All of the experiments were conducted in triplicate and the results were presented as mean with standard error mean (SEM). Student t-test was used to determine statistical significance, with a *P*-value of less than 0.05 considered significant.

## Results

### Overexpression of PIN1 in NPC primary tumors and tumor lines

**Fig 1:**
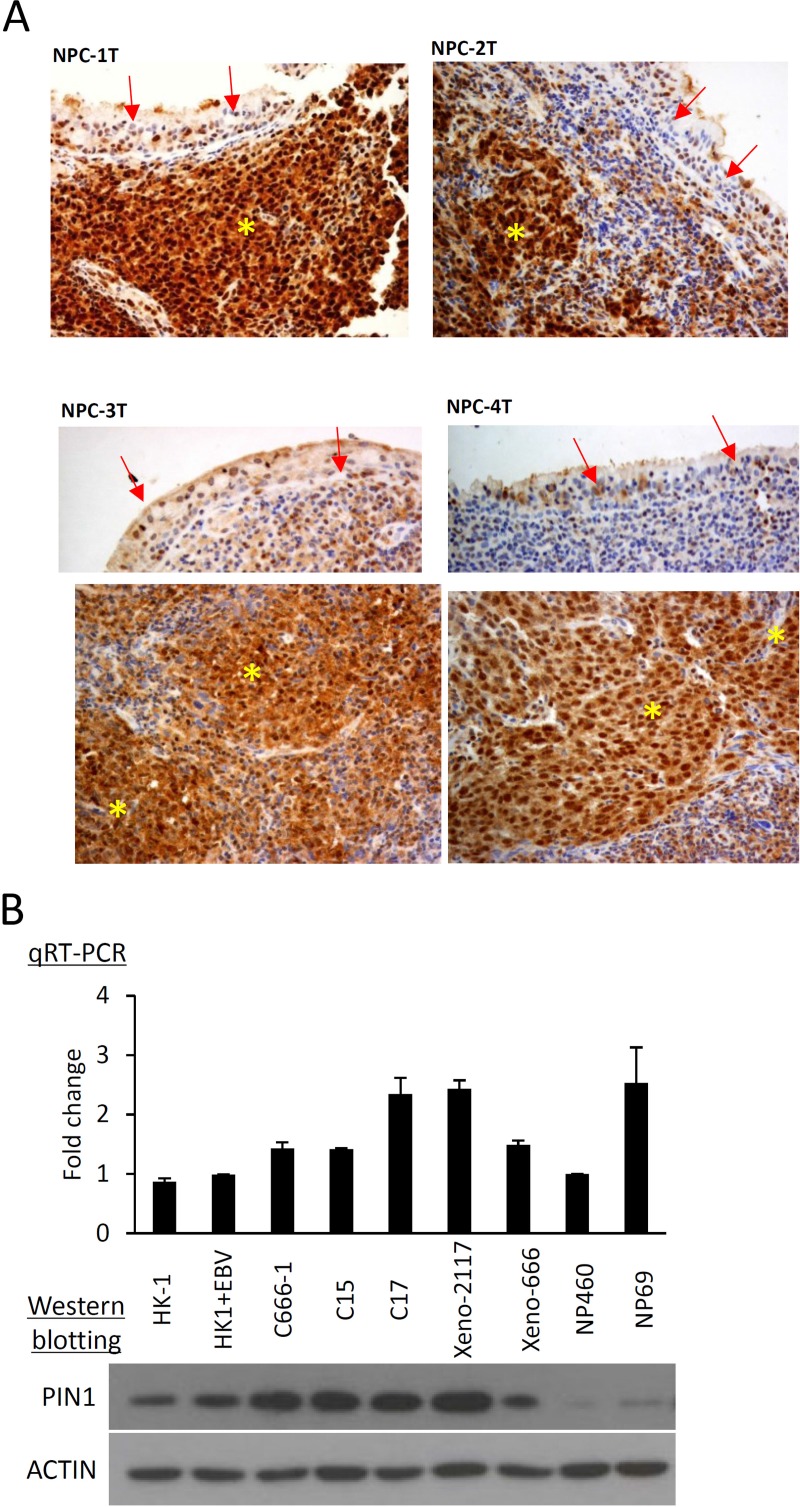
Overexpression of PIN1 in EBV-associated NPC. (A) IHC staining was used to illustrate the overexpression of PIN1 in representative NPC primary tumors (NPC1-NPC4). All of the cases were EBV-positive undifferentiated carcinomas. NPC1, NPC3 and NPC4 are from the patients with stage 3 disease. NPC2 is from a patient with stage 2 NPC. Strong PIN signals were found in the tumor cells, which are indicated by yellow “_*_”. The adjacent normal epithelium served as the control in which weak PIN1 signals were observed. The red arrows indicate normal nasopharyngeal epithelium. (B) Expression of PIN1 in NPC tumor lines, xenografts and immortalized normal NP cells, detected by qRT-PCR (upper panel) and Western blot (lower panel). For qRT-PCR, the *PIN1* transcription in NP460 was used as a reference. The relative expression of *PIN1* transcripts was indicated as a fold difference over the reference. β-actin was used for loading normalization. Similarly, ACTIN was used as an internal loading control in the Western blot analysis. The weak PIN1 expression in NP69 and NP460 suggested that the downregulation of PIN1 involved post-translational regulation.

Using immunohistochemical staining, we detected the overexpression of PIN1 in 70/70 (100%) NPC primary tumors, compared with the adjacent normal nasopharyngeal epithelium (Fig 1A). PIN1 overexpression was also demonstrated in a panel of EBV-positive NPC cell line (C666-1) and xenografts (C15, C17, xeno-2117 and xeno-666) by Western blotting, whereas only weak PIN expression was detected in the immortalized normal nasopharyngeal epithelial cell lines (NP460 and NP69) (Fig 1B). Despite the dramatic reduction in PIN1 protein expression in the immortalized normal NPC cells, no obvious differences in *PIN1* mRNA transcriptions were observed between the normal NP cell lines and the NPC tumors. This finding suggests a post-translational regulation of PIN1 in normal nasopharyngeal cells.

To determine the mechanism of post-translational regulation on PIN1 proteins, HK-1 and NP69 were treated with the proteasome inhibitor MG132 (Carbobenzoxy-Leu-Leu-leucinal). The Western blot results revealed increased PIN1 protein expression in both HK1 and NP69 cells after MG132 treatment (S1 Fig). This indicates that PIN1 protein expression is regulated by proteasome degradation.

The consistent overexpression of PIN1 suggests its potential oncogenic role in NPC development. To investigate its oncogenic function, siRNAs and the PIN1 inhibitor Juglone were used to determine the effect of PIN1 suppression on NPC cell growth. Subsequently, the effects of PIN1 expression on cell growth and tumorigenesis were examined in normal nasopharyngeal epithelial cells (NP69) transfected with a PIN1 expressing vector.

### PIN1 regulates NPC cell growth and cyclin D1 expression

**Fig 2:**
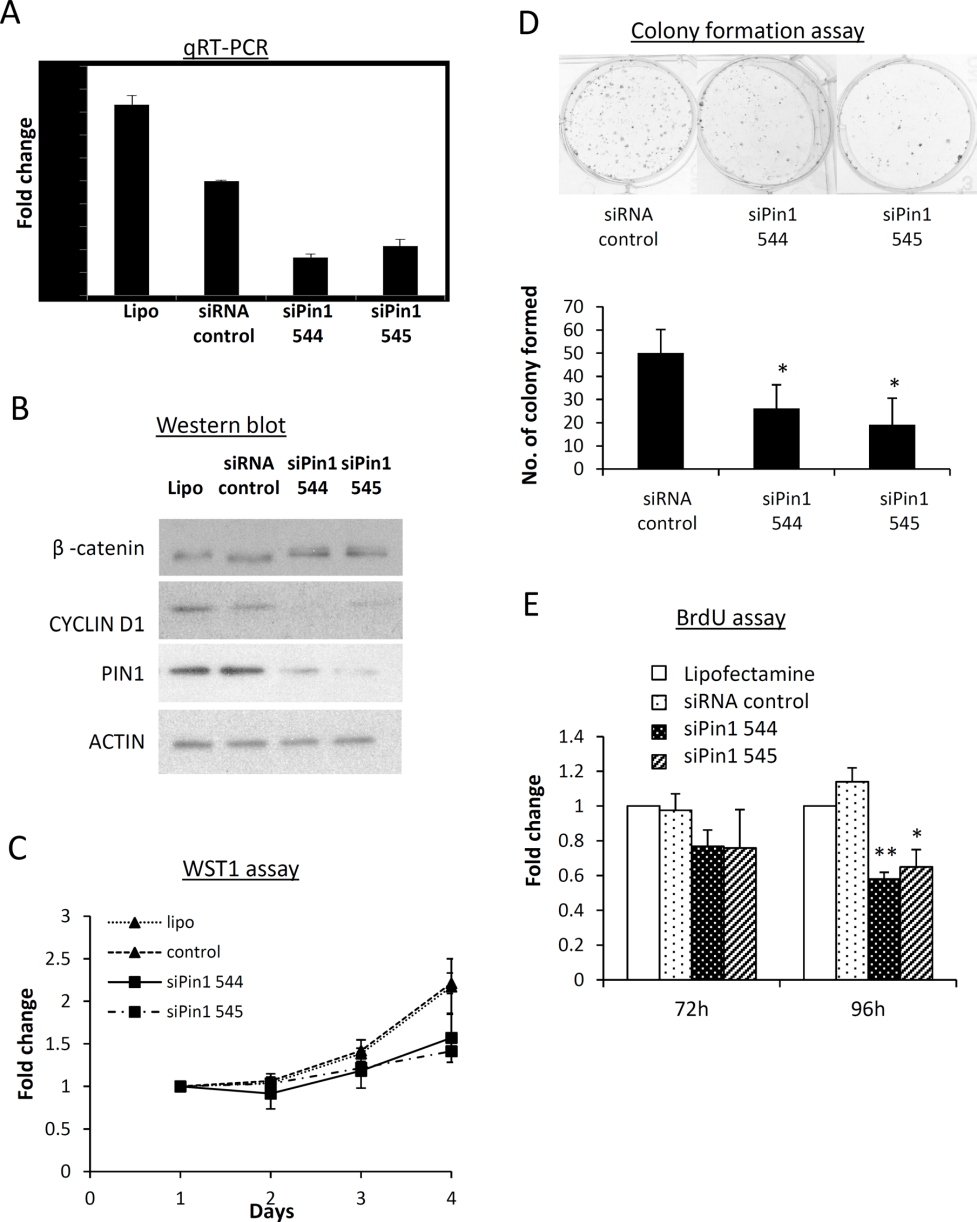
**PIN1 suppression inhibits cell growth, DNA synthesis, colony formation ability and cyclin D1 expression in NPC cells** (A) qRT-PCR and (B) Western blot were used in the downregulation of PIN1 transcription and protein expression in PIN1 siRNAs (siPIN1 544 and siPIN1 545)-treated NPC cells and C666-1, respectively. In these PIN1 knockdown cells, reduced cyclin D1 expression was observed. ACTIN was used as the loading control in the Western blot analysis. (C) WST-1 assay revealed growth inhibition in the NPC cells transfected with siPin1 544 and siPin1 545. (D) Colony formation ability was significantly suppressed in PIN1-silenced C666-1 cells. Representative photos of colonies formed by siRNA-treated and control cells are shown. Statistical significance was determined by Student t-test, where a *P*-value of less than 0.05 was considered significant (*P < 0.05, **P < 0.01). (E) A BrdU assay was used to reveal the significant inhibition of DNA synthesis in the PIN knockdown NPC cells.

The effect of PIN1 knockdown on NPC cell growth was investigated by transfecting C666-1 cells with PIN1-specific siRNAs (siPin1 544 and siPin1 545). Fig 2A and 2B show the marked downregulation of *PIN1* mRNA and protein in the C666-1cells transfected with siPIN1. The results of the WST-1 assay demonstrated observable growth inhibition in the siPIN1-transfected C666-1 cells, compared with the control (Fig 2C). Furthermore, the colony formation ability was significantly reduced in the siPIN1-transfected C666-1 (Fig 2D). These findings indicate that PIN1 expression regulates NPC cell growth. The results of the BrdU assay revealed significant suppression of DNA synthesis in the PIN1 knockdown NPC cells (Fig 2E). Importantly, the expression of cell cycle regulator cyclin D1 was repressed in the PIN1 knockdown C666-1 cells (Fig 2B). The co-transfection of pCMV-cyclinD1, a cyclin D1-expressing vector with siPIN1, restored the cyclin D1 expression, cell proliferation, DNA synthesis and colony formation ability in C666-1 cells compared with those of siRNA-treated cells (S2 Fig). This suggests that PIN1 regulates NPC cell growth by modulating cyclin D1 expression.

### PIN1 inhibitor induces caspase-3 and reduces tumor size

**Fig 3:**
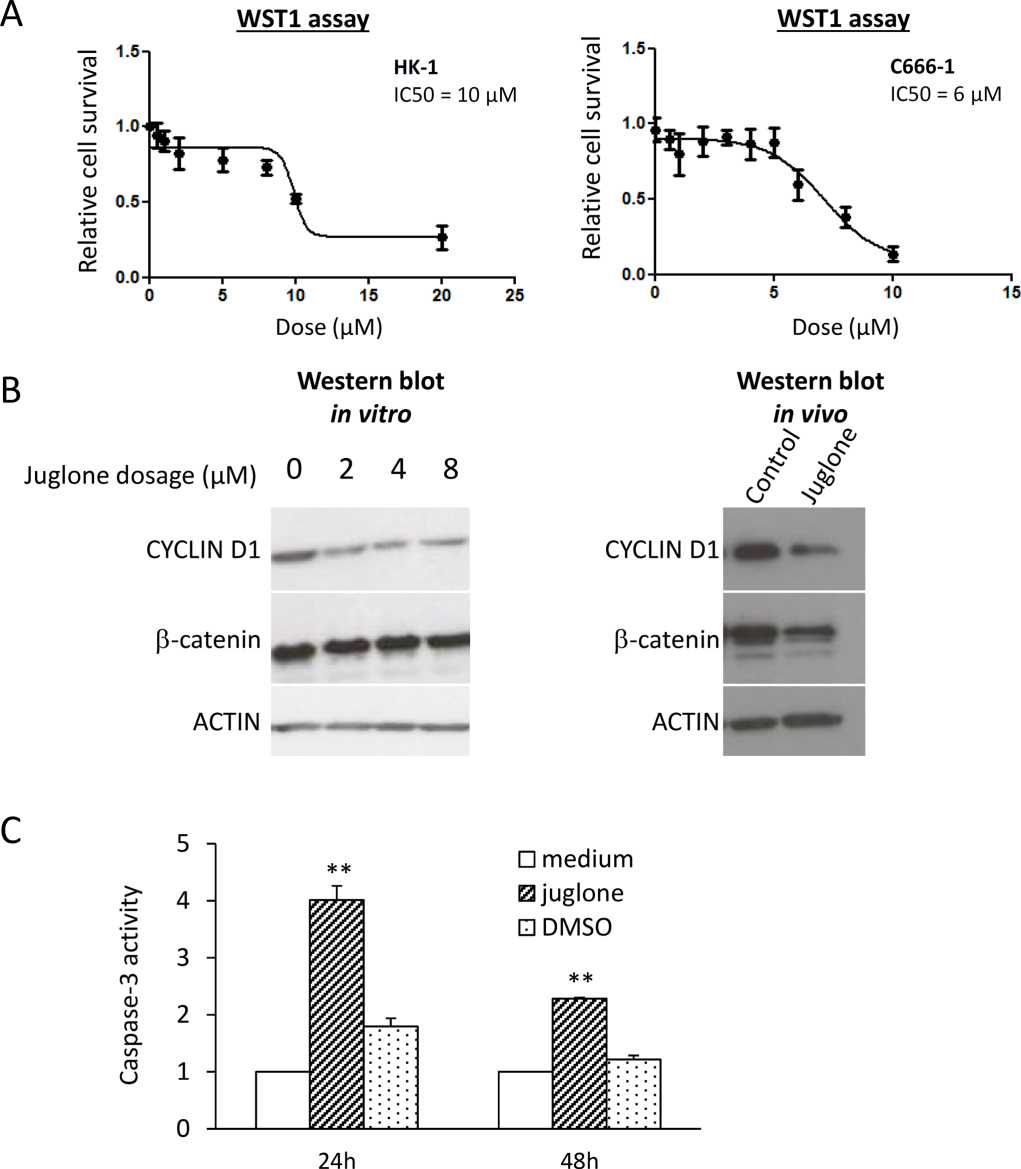
PIN1 inhibitor Juglone suppresses tumor cell growth and induces caspase-3 activity. (A) HK-1 (left panel) and C666-1 (right panel) were treated with the PIN1 inhibitor Juglone for 24 hours to determine the drug dose sensitivity. The IC50 values of the C666-1 and HK1 cells were 6 μM and 10 μM, respectively. (B) The expression of cyclin D1 was suppressed by Juglone in a dose-dependent manner, but no significant changes in β-catenin expression were detected. The expression of cyclin D1 and β-catenin in the Juglone-treated C-666 xenograft model was examined by Western blot. Reduced expression of cyclin D1 was observed in the tumor that had received Juglone treatment. PIN1 inhibition resulted in suppression of cyclin D1 both *in vitro* and *in vivo*, and there was a modest reduction in the β-catenin level in the *in vivo* model. (C) The C666-1 cells exhibited significantly higher caspase-3 activity after Juglone treatment, compared with their untreated or DMSO-treated counterparts. This indicates that PIN1 inhibition can activate the caspase apoptotic pathway in NPC cells. Statistical significance was determined by Student t-test, *P*-value of less than 0.05 was considered significant (*P < 0.05, **P < 0.01).

A PIN1 inhibitor, Juglone, was used to elucidate the effects of PIN inhibition in NPC cells *in vitro* and *in vivo*. A WST-1 assay showed that the half maximal inhibitory concentration (IC50) of Juglone for C666-1 and HK1 was 6 and 10 μM, respectively (Fig 3A). This finding indicates that C666-1 cells with high PIN1 expression are more susceptible to Juglone treatment. The PIN1 inhibitor Juglone suppressed the cyclin D1 expression in C666-1 cells (Fig 3B, left panel). The *in vivo* effect of the PIN1 inhibitor on cyclin D1 repression was further confirmed by the intra-tumor injection of Juglone into C666-1 xenografts in nude mice models (Fig 3B, right panel). Juglone treatment also significantly enhanced caspase-3 activity in C666-1 cells, compared with the controls (Fig 3C). The results suggest that Juglone inhibits cell growth and induces apoptosis in NPC cells.

**Fig 4:**
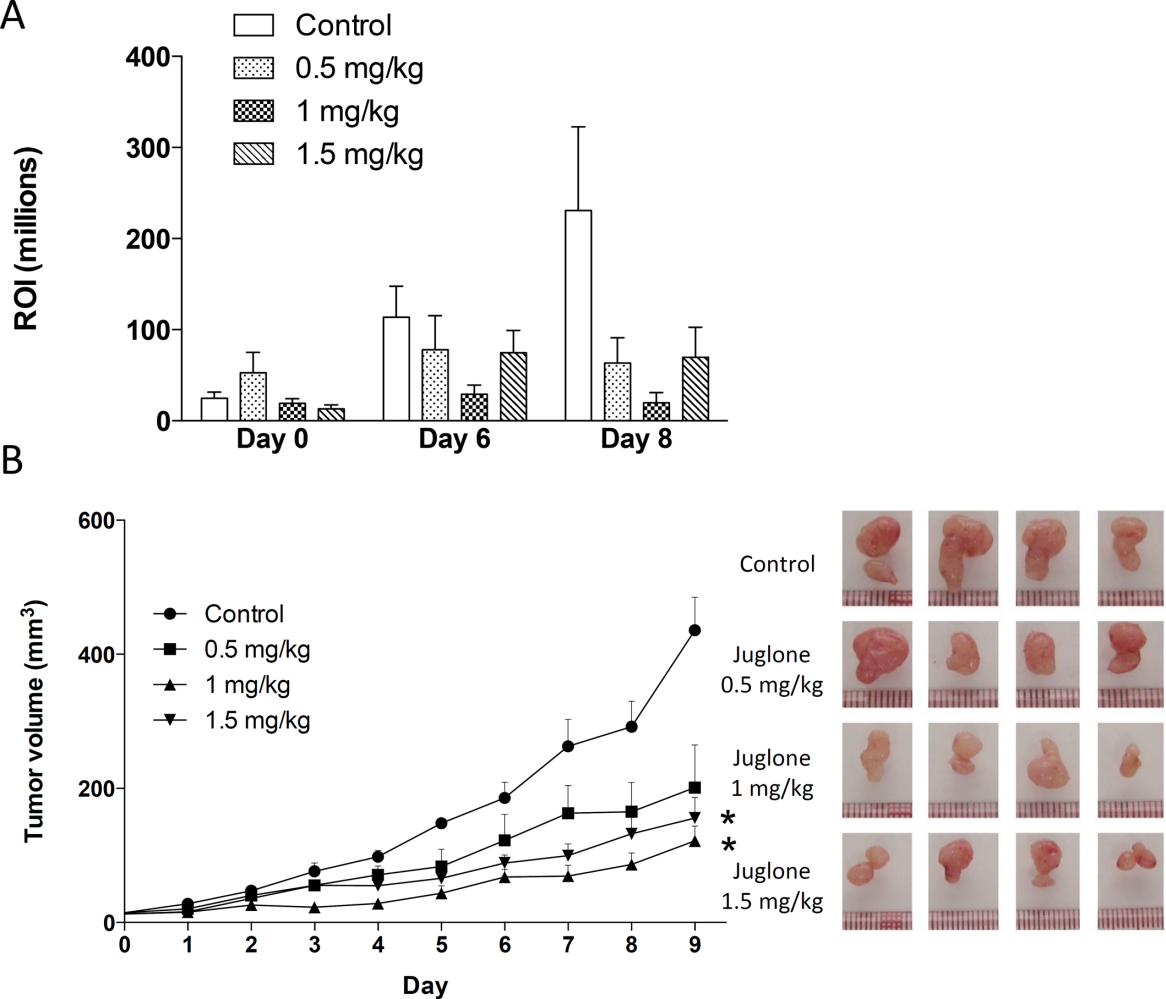
PIN1 inhibitor Juglone suppresses tumor growth *in vivo*. (A) Using *in vivo* imaging, the tumor growth of luciferase-tagged C666-1 was revealed after treatment with different dosages of Juglone (ROI counts per million photons per second). The luciferase signal was increased in the control untreated tumors during the Juglone treatment. In the mice treated with Juglone in different doses (0.5, 1 and 1.5 mg/kg), consistently lower luciferase signals were observed during the 8-day treatment periods. (B) Significant inhibition of tumor growth was shown in the mice treated with 0.5, 1 and 1.5 mg/kg Juglone. Four nude mice were used in each study group. Statistical significance was determined by Student t-test, where a *P*-value of less than 0.05 was considered significant (*P < 0.05, **P < 0.01).

Fig 4A and 4B demonstrate the *in vivo* antitumor effect of Juglone in NPC cells. The ROI count was highly reduced in the mice treated with Juglone, compared with that of the controls (Fig 4A). As Fig 4B shows, the tumor sizes in the mice treated with Juglone were significantly reduced, compared with the control (Fig 4B). A significant inhibition of tumor growth was observed in mice treated with 1 mg/kg and 1.5 mg/kg Juglone.

### PIN1 induces anchorage-independent growth of nasopharyngeal epithelial cells

**Fig 5:**
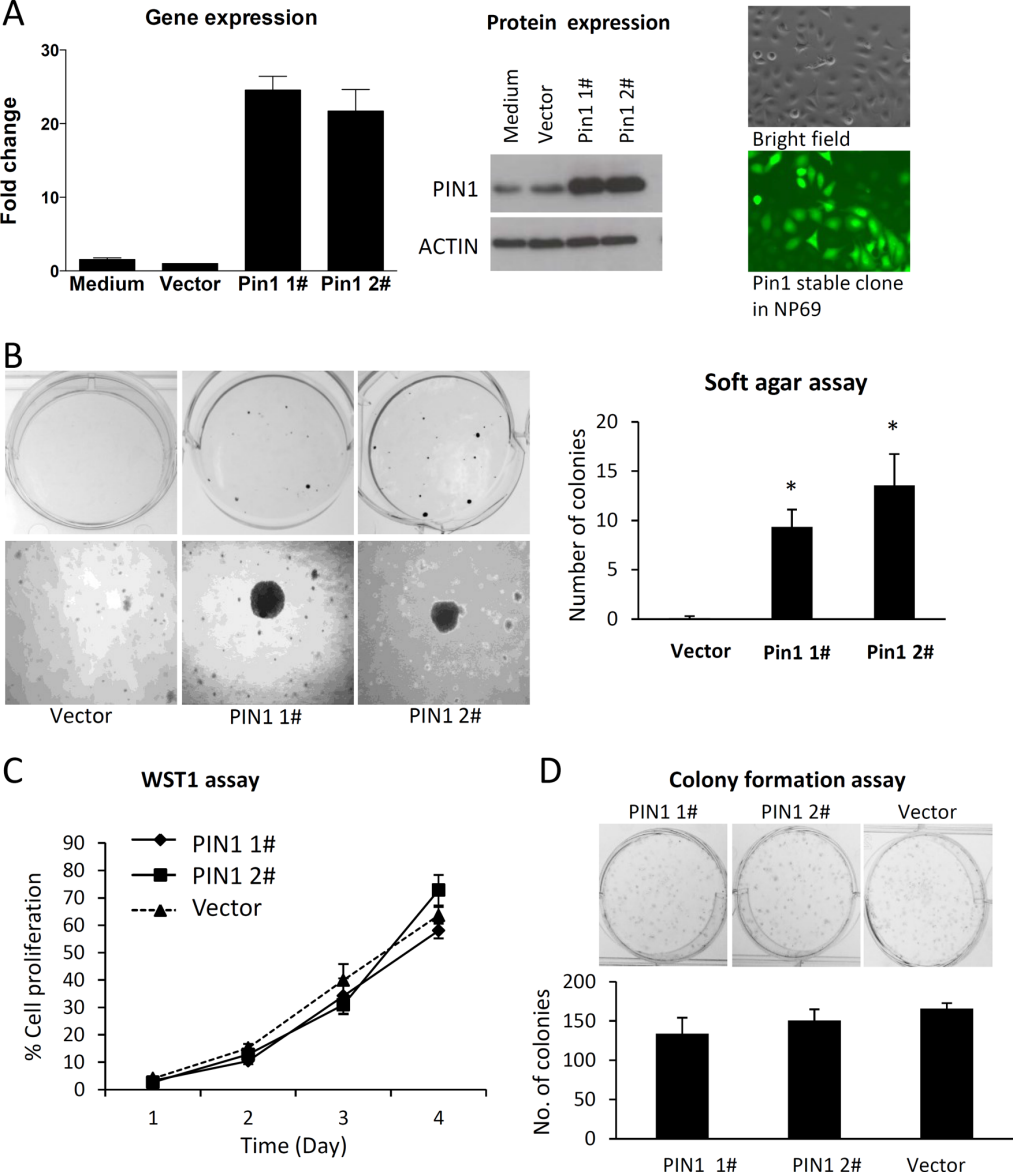
PIN1 overexpression enhances anchorage-independent growth in nasopharyngeal epithelial cells. (A) The overexpression of PIN1 was detected in NP69 cells transfected with Pin1-expressing lenti-viral vectors (Pin1 1# and Pin1 2#) by qPCR (left panel) and Western blot (middle panel). Representative photos of PIN1-transfected cells by bright field (right upper panel) and fluorescence field (right lower panel) are shown. The transfection efficiency was monitored by GFP expression. (B) Enhanced anchorage-independent growth on soft agar in NP69 cells with PIN1-overexpression, compared with control cells (left upper panel, colonies in 6-well plates; left lower panel, colonies under microscope; right panel, average data in histogram). (C-D) No significant effect of PIN1 overexpression on cell growth and colony formation ability was detected in the immortalized nasopharyngeal epithelial cells NP69, by WST-1 and colony formation assay, respectively. Statistical significance was determined by Student t-test, where a *P*-value of less than 0.05 was considered significant (*P < 0.05).

The tumorigenic effect of PIN1 overexpression was further studied in an immortalized nasopharyngeal epithelial cell line (NP69) transfected with two PIN1 mimics (pCDH-CMV-MCS-EF1-copGFP-PIN1/“Pin1 1#” and “Pin1 2#”). The overexpression of *PIN1* transcripts and proteins was observed in the PIN1-transfected NP69 cells (Fig 5A). Fig 5A shows the high transfection efficiency of PIN1-transfected cells expressing GFP.

The overexpression of PIN1 in N69 cells significantly induced anchorage-independent cell growth in the soft agar assays (Fig 5B). The number of colonies formed in soft agar by PIN1-expressing NP69 cells was significantly higher than those of vector control. However, PIN1 overexpression did not induce cell proliferation and colony formation ability in NP69 cells (Fig 5C and 5D).

### PIN1 overexpression activates MAPK/JNK pathway

**Fig 6:**
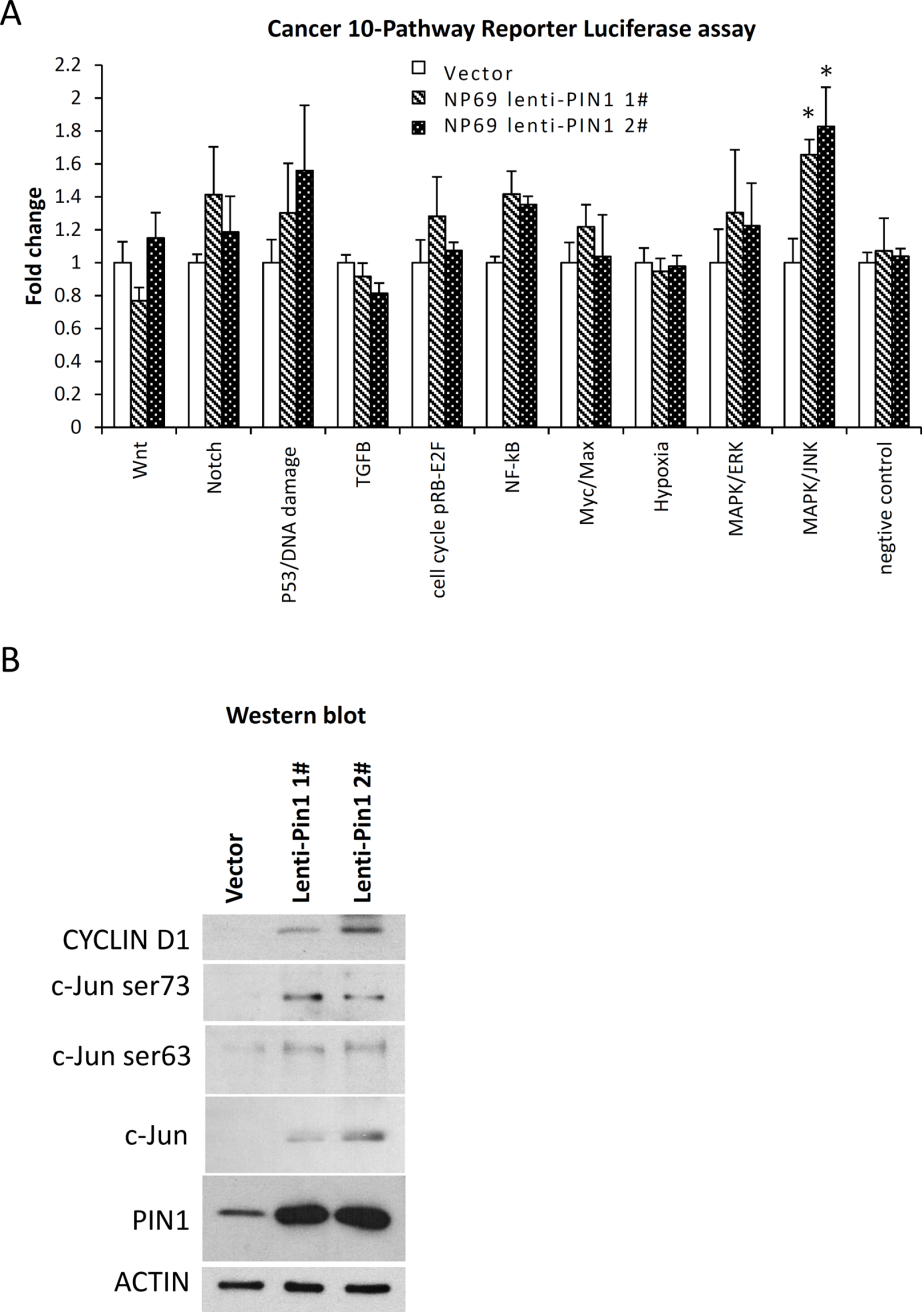
Overexpression of PIN1 activates the MAPK/JNK pathway. (A) Using the Cancer 10-Pathway Reporter Luciferase assay, significant activation of the MAPK/JNK signaling pathway was observed in two stably PIN1-overxpressing NP69 cell lines (NP69lenti-PIN1 1# and NP69lenti-PIN1 2#). For these PIN1-expressing cells, the activity was also moderately activated in the NOTCH, P53/DNA damage and NF-κB pathways. (B) Western blot analysis demonstrated the upregulation of c-Jun, phosphorylated c-Jun (Ser63 and Ser73) and cyclin D1 in the stable PIN1-transfected NP69 cells. Statistical significance was determined by Student t-test, where a *P*-value of less than 0.05 was considered significant (*P < 0.05).

A reporter luciferase assay revealed that the MAPK/JNK signaling pathway was significantly activated in NP69 cells stably expressing PIN1, compared with those transfected with a vector (Fig 6A). The activation of the NOTCH, p53 and NF-κB pathways was also observed in PIN1-expressed NP69 cells, although it did not reach statistical significance. Aside from cyclin D1, Western blotting confirmed the upregulation of both c-Jun and phosphoryl-c-Jun (Ser63 and Ser73) in NP69 cells stably transfected with PIN1 (Fig 6B). This finding suggests that PIN1 induces the growth of nasopharyngeal epithelial cells by activating the MAPK/JNK pathway.

## Discussion

In a recent study, Lu *et al*. demonstrated the association of PIN1 promoter polymorphisms and risk of NPC [22]. In the current study, we provide the functional evidence on the importance of PIN1 overexpression in NPC tumorigenesis. We show that PIN1 suppression inhibits *in vitro* and *in vivo* tumor growth in NPC cells while its overexpression induces the anchorage-independent growth of nasopharyngeal epithelial cells. The findings suggest that PIN1 overexpression contributes to NPC tumorigenesis, and that its inhibition may be a potential therapeutic strategy for EBV-associated NPC.

PIN1 is an important enzyme that binds phosphorylated Ser/Thr-Pro motifs and catalyzes the cis/trans-isomerization of proline-containing peptides [5]. The overexpression of PIN has been reported in various human tumor types, including breast [23], esophageal [24], prostate [25] and colorectal cancer [26, 27]. In this study, constitutive PIN1 overexpression was found in EBV-associated NPC tumor cells, but PIN1 protein was scarcely detected in EBV-negative nasopharyngeal epithelial cells. Since *PIN1* transcription is similar in both NPC and normal NP cells, the PIN overexpression in the tumor cells is regulated by post-transcriptional mechanisms. A recent study reported that PIN1 expression is regulated by the tumor suppressive miRNA miR-296-5p in prostate cancer [28]. However, aberrant mir-296-5p expression is rarely detected in NPC [29]. The restoration of PIN expression in the MG132-treated NP cells suggests that the process of proteasomal degradation is an important mechanism in regulating PIN1 function. Basu *et al*. showed that the proteasomal degradation of PIN1 was crucial in its interaction with BCL2 and tumor cell survival [30]. Together with the current findings, the lack of or inadequate proteasomal degradation may be a potential mechanism in explaining PIN1 accumulation in EBV-associated NPC, which subsequently aids tumor cell survival. In our recent study, we revealed the interaction of EBV latent protein EBNA1 with PIN1 in NPC cells (unpublished data). Nevertheless, the binding did not inhibit the proteasomal degradation PIN1. The mechanisms for PIN1 overexpression in NPC cells needs to further elucidate in future studies.

PIN1 is crucial in tumor cell transformation, as it activates oncogenic pathways and growth enhancers [31] while inactivating tumor suppressors and growth inhibitors [32, 33]. Among the PIN1-activated oncogenes, Cyclin D1 is of particular importance in NPC tumorigenesis. The overexpression of cyclin D1 has been detected in more than 90% of primary NPC tumors. In NPC cells, cyclin D1 not only plays an essential role in cell proliferation but its overexpression can stabilize EBV infection [34]. PIN1 is thought to play an important role in NPC pathogenesis by regulating cyclin D1 expression. Although there is no significant effect of PIN1 overexpression on cell proliferation and colony formation in normal epithelial cells (Fig 5C), cyclin D1 is up-regulated by PIN1 expression. PIN1 overexpression may promote EBV infection in nasopharyngeal epithelial cells via cyclin D1 upregulation as we reported previously [34]. Further studies are needed to determine the role of PIN1 in the enhancement of EBV infection and the maintenance of the stable latent EBV genome in nasopharyngeal epithelial cells—the critical steps in NPC tumorigenesis.

PIN1 has been shown to regulate cyclin D1 via the JNK, WNT or NF-κB pathways. As Fig 6A and 6B show, PIN1 significantly activated the MAPK/JNK pathway and induced cyclin D1 expression in NP69 cells. It is probable that PIN1 modulates cyclin D1 expression by activating the JNK pathway. Cyclin D1 expression can be induced by the interaction between PIN1 and the p-Ser63/73-Pro motifs in Jun, which positively regulates Jun transcriptional activity on its target genes. Our findings concur with those of studies on human breast cancer in which PIN1 overexpression resulted in increased JNK activity [35]. In addition to JNK, PIN1 can also modulate cyclin D1 expression via the β-catenin of the WNT pathway [36]. PIN1 overexpression has also been shown to up-regulate cyclin D1 and β-catenin in hepatocellular carcinomas (HCC) [37]. However, PIN1 expression did not significantly up-regulate the β-catenin expression in NPC cells, and thus the WNT pathway might not be involved in PIN1-modulated cyclin D1 expression. It is well established that the NF-κB and NOTCH pathways are critically activated pathways in NPC [38–40]. PIN1 has been reported to target the pThr254-Pro motif in NFκB1 and p65 [41], which inhibits p65 from binding to its inhibitor IκB, resulting in the protein nuclear accumulation and stability of these proteins. Our study confirms that PIN1 overexpression can up-regulate the NF-κB, NOTCH and p53 pathways. Further studies are required to fully elucidate the role of PIN1 in regulating these cancer-related signaling pathways in NPC. The current study also reveals the role of PIN1 in inducing anchorage-independent cell growth and cyclin D1 expression in normal nasopharyngeal epithelial cells (Fig 5B), suggesting that PIN1 is likely to play an important role in NPC tumorigenesis. The implication of PIN1 in carcinogenesis has also been demonstrated in HCC [37].

Regarding the tumorigenic properties of PIN1, we evaluated the effects of the PIN1 inhibitor Juglone on NPC tumor cell growth, apoptosis and *in vivo* tumor development. Juglone is a natural component of the Juglans mandshurica Maxim that was previously proven to be a potent cytotoxic agent in human tumor cell lines by exerting multiple anti-tumor activities such as cell apoptosis [42–44]. Apoptosis is a well-controlled type of cell death, and its induction has proven a useful approach in cancer therapies [45–47]. Our study confirmed that Juglone significantly induced caspase-3 activity and inhibited tumor growth *in vivo*. Concordant with our PIN1 siRNA study, the depletion of PIN1 suppresses tumor cell proliferation, DNA synthesis and colony formation in NPC cells. Previous studies have shown that PIN1 inhibition can suppress the Neu- and Ras-induced transformed phenotypes, in addition to inducing mitotic arrest and apoptosis in breast cancer cells. Moreover, PIN1 knockout mice have demonstrated aberrant cell proliferation that resulted in various abnormalities such as retinal degeneration, neurological abnormality and testicular atrophy [48]. The increase in caspase-3 activity via Juglone suggested that it might induce tumor cell apoptosis and inhibit tumor formation in the nude mice model. Our findings indicate that Juglone might serve as a potential anti-tumor drug for NPC patients.

## Conclusions

Our study provides evidence supporting the oncogenic role of PIN1 in NPC tumorigenesis. The overexpression of PIN1 might enhance tumor cell growth via the upregulation of cyclinD1. Thus, PIN1 inhibition serves as a potential therapeutic approach for NPC patients.

## Supporting Information

S1 Fig Proteasomal degradation of PIN1 in nasopharyngeal epithelial cells. Using Western blot, elevated PIN1 proteins were observed in the nasopharyngeal epithelial cells, NP69 and HK-1, after treatment with proteasome inhibitor MG132 (0–15 μM). ACTIN was used for loading normalization.
(TIF)

S2 Fig PIN1 enhances NPC cell growth through cyclin D1 induction. (A) The expression of PIN1 and cyclin D1 in NPC C666-1 cells transfected with PIN1 siRNAs and cyclin D1-expressing vectors was observed via Western blot. Using (B) WST-1, (C) BrdU and (D) colony formation assays, the expression of cyclin D1 was shown to restore the cell growth and DNA synthesis in the PIN1 knockdown NPC cells.
(TIF)
