# Supplementary figures and images for: Overexpression of PIN1 Enhances Cancer Growth and Aggressiveness with Cyclin D1 Induction in EBV-Associated Nasopharyngeal Carcinoma
Source: PLoS One. 2016 Jun 3;11(6):e0156833. doi: 10.1371/journal.pone.0156833 (PMC4892693; doi:10.1371/journal.pone.0156833)

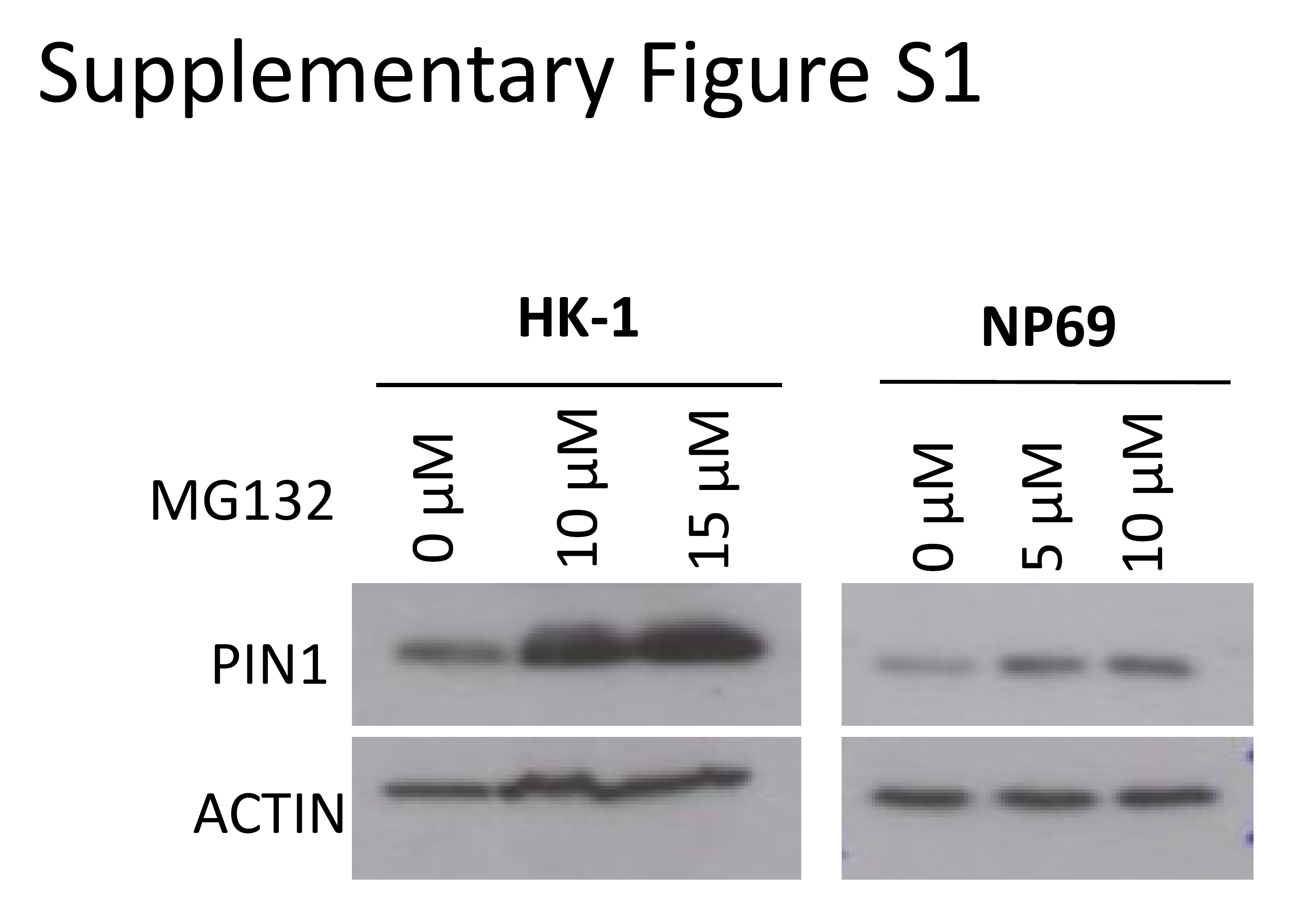

Supplement: S1 Fig — Using Western blot, elevated PIN1 proteins were observed in the nasopharyngeal epithelial cells, NP69 and HK-1, after treatment with proteasome inhibitor MG132 (0–15 μM). ACTIN was used for loading normalization. (TIF) [file pone.0156833.s001.tif]

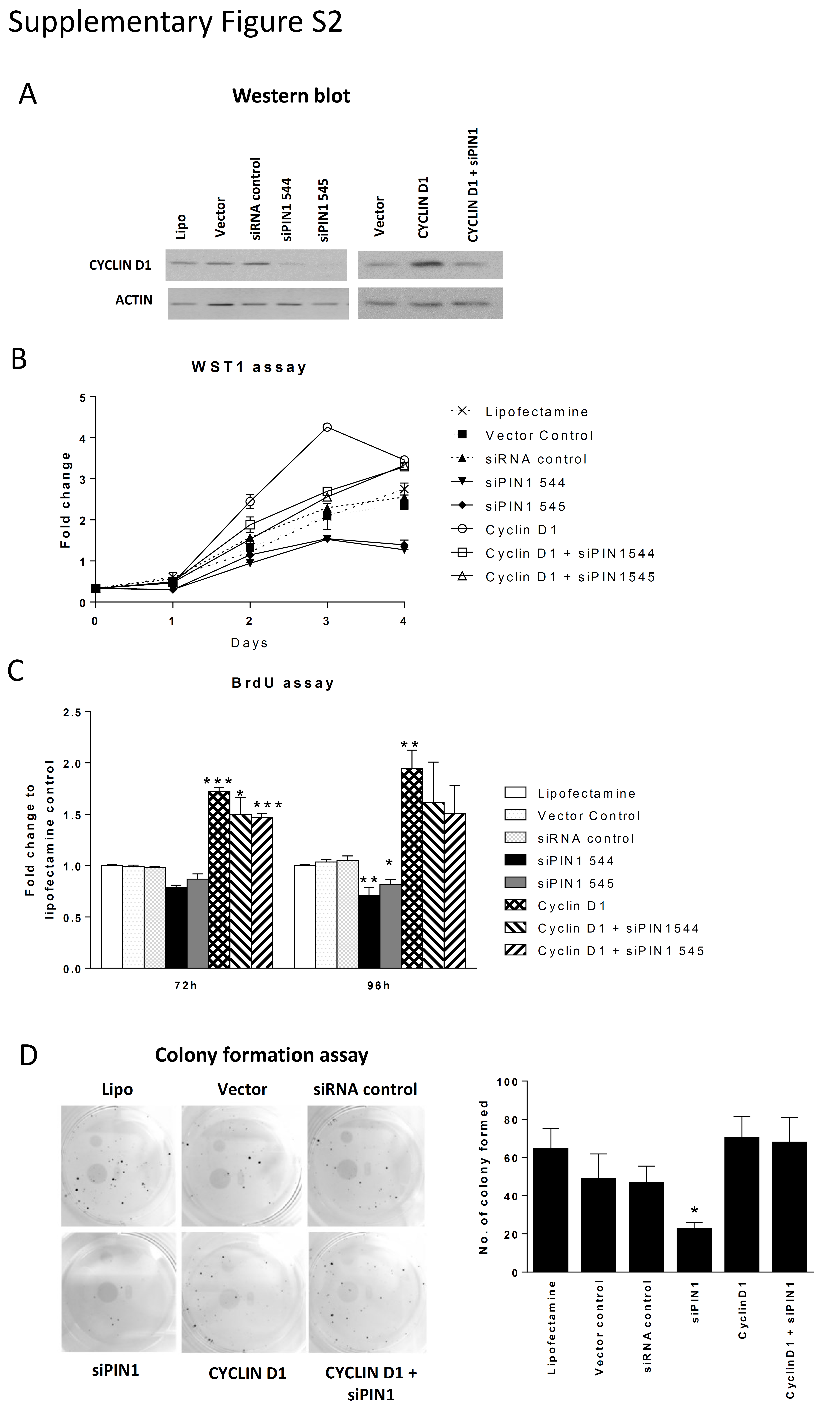

Supplement: S2 Fig — (A) The expression of PIN1 and cyclin D1 in NPC C666-1 cells transfected with PIN1 siRNAs and cyclin D1-expressing vectors was observed via Western blot. Using (B) WST-1, (C) BrdU and (D) colony formation assays, the expression of cyclin D1 was shown to restore the cell growth and DNA synthesis in the PIN1 knockdown NPC cells. (TIF) [file pone.0156833.s002.tif]
